# HHV-8-Associated Lymphoproliferative Disorders and Pathogenesis in an HIV-Positive Patient

**DOI:** 10.1155/2019/4536157

**Published:** 2019-08-22

**Authors:** Carlo Guerrero, Tania Jain, Katalin Kelemen

**Affiliations:** ^1^Department of Medicine, Houston Methodist Hospital, Houston, TX, USA; ^2^Adult Bone Marrow Transplantation Service, Memorial Sloan Kettering Cancer Center, New York, NY, USA; ^3^Department of Laboratory Medicine and Pathology, Mayo Clinic, Phoenix, AZ, USA

## Abstract

Human herpesvirus 8 (HHV-8), also known as Kaposi's sarcoma-associated herpesvirus, is a DNA oncovirus known for its role in the development of Kaposi's sarcoma (KS) and several lymphoproliferative disorders (LPDs). HHV-8 promotes lymphoproliferation via the activation of the interleukin-6 receptor signaling pathway, as well as a host of other regulatory mechanisms. The spectrum of HHV-8-associated LPDs is increasing. The World Health Organization has recently updated the classification of HHV-8-associated LPDs by introducing HHV-8-positive germinotropic LPD (GLPD) in addition to the previously recognized entities of HHV-8-positive diffuse large B-cell lymphoma, not otherwise specified (DLBCL, NOS), primary effusion lymphoma (PEL), and HHV-8-positive multicentric Castleman's disease (MCD). We present here a case of an HIV-positive woman with a history of KS, who later developed three HHV-8-associated LPDs, including HHV-8-positive MCD, PEL, and GLPD. To the best of our knowledge, this is the first reported case of a patient with this combination of individually rare HHV-8-associated LPDs. This case illustrates the spectrum and the sequential development of the different clinical manifestations of HHV-8-associated diseases. Detection of HHV-8 can have clinical significance in the diagnosis and management of certain HHV-8-associated conditions. Recently discovered variants of HHV-8-associated LPDs indicate that this group represents a diverse spectrum of disorders, whose classification may require further refinement beyond the currently recognized entities.

## 1. Introduction

The first description of Kaposi's sarcoma (KS) was made in 1872 by the eponymous dermatologist Moritz Kaposi [1]. He documented what we now characterize as the “classic” form of KS, slow-growing skin tumours seen predominantly in elderly men of Mediterranean or Jewish ancestry [2]. More than a century later, the epidemic of human immunodeficiency virus (HIV) in North America unleashed a more aggressive form of KS associated with the acquired immunodeficiency syndrome (AIDS). This led to the discovery of human herpesvirus 8 (HHV-8), also known as Kaposi's sarcoma-associated herpesvirus, as the causative infectious agent of KS [3].

Since the discovery of its link to KS, HHV-8 has been recognized to be associated with several lymphoproliferative disorders (LPDs), including HHV-8-positive multicentric Castleman's disease (MCD), primary effusion lymphoma (PEL), HHV-8-positive germinotropic lymphoproliferative disorder (GLPD), and HHV-8-positive diffuse large B-cell lymphoma, not otherwise specified (DLBCL, NOS). The 2016 update of the WHO Classification of Tumours of Haematopoietic and Lymphoid Tissues included HHV-8-positive GLPD as a newly classified entity within the category of HHV-8-associated LPDs [4]. These HHV-8-associated LPDs constitute a spectrum of disorders which can be distinguished from one another based on their clinical manifestations, microscopic appearance, localization, immunophenotype, genetic profile, and the presence of an Epstein–Barr virus (EBV) coinfection. However, there have been reported cases that have overlapping features of several of these HHV-8-associated LPDs, and which may also manifest clinically in ways distinct from these WHO-classified disorders [5–7].

We present a case of an HIV-positive woman who developed KS, HHV-8-positive MCD, PEL, and GLPD. Through this case report, we aim to illustrate the pathologic spectrum of three HHV-8-associated LPDs and highlight the role that HHV-8 plays in the development of its associated LPDs, including a discussion of key mechanisms by which it promotes cellular proliferation. We emphasize the long-term effects of HHV-8 infection in the immunocompromised host, resulting in sequential development of several different LPDs in the same patient over time. Finally, we discuss several fundamental questions that arise from this case regarding the spectrum of HHV-8-associated LPDs.

## 2. Case

The patient is an African-American woman originally from Zimbabwe, who moved to the United States in 2007 at the age of 41. She was diagnosed with HIV/AIDS and KS in 2001 and was treated at that time with combination antiretroviral therapy (ART). Her medical records from Zimbabwe were not available for review. She established medical care in the United States in 2007. In late 2007, she was diagnosed with new KS lesions and HHV-8-positive MCD. Her CD4 count was 162 cells/*μ*L, and her HIV viral load was 256 copies/mL. She had associated anemia, hypoalbuminemia, and elevated sedimentation rate. She was treated with rituximab and vincristine from 2008 to 2013, with intermittent interruptions in treatment due to cytopenias.

**Figure 1:**
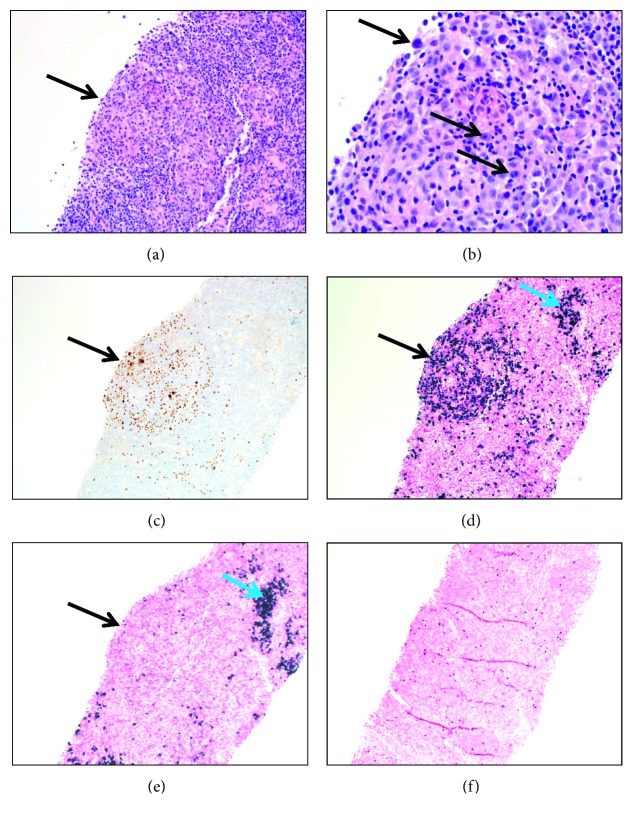
Pathologic findings in an inguinal lymph node core biopsy. (a) Germinal center showing large atypical plasmablasts. The arrow indicates the germinal center (H&E, 200x). (b) Germinal center, arrows point to plasmablasts (H&E, 500x). (c) The plasmablasts (arrow) are positive for HHV-8 (immunostaining, anti-HHV-8 (13b10) mouse monoclonal antibody by Cell Marque, 200x). (d, e) The germinal center (black arrows) contains lambda-light chain-restricted plasmablasts (in situ hybridization, DNA probes by Ventana that react with lambda mRNA and Kappa mRNA, respectively, 200x). The paracortex adjacent to the germinal center contains polyclonal plasma cells (blue arrows). (f) EBV-positive cells throughout the lymph node core biopsy (EBER in situ hybridization, DNA probe to EBV-encoded RNA-1 by Ventana, 100x).

In January 2014, she presented to our institution for a sepsis-like illness and worsening diffuse lymphadenopathy. Her infectious workup was unrevealing. Her CD4 count was 307 cells/*μ*L, and her HIV viral load was undetectable (i.e., <20 copies/mL). A core biopsy of an enlarged right inguinal lymph node was performed. The morphologic findings were consistent with the patient's known diagnosis of MCD, plasmacytic type. The lymph node architecture was preserved, and there was dense polyclonal plasma cell proliferation filling the paracortex. HHV-8 latency-associated nuclear antigen (LANA) immunostain was positive, with virus-infected cells localized primarily within germinal centers. Occasional germinal centers stood out due to the replacement of the germinal centers by large plasmacytoid cells (i.e., plasmablasts). Some of these atypical germinal centers showed lambda-light chain restriction of the plasmablasts, meeting the criteria of HHV-8-positive GLPD (Figure 1). EBV-encoded small RNA (EBER) in situ hybridization was positive for EBV. Her presenting signs and symptoms were ultimately attributed to progression of MCD, especially since she had not received vincristine treatment for six weeks prior to presentation due to severe cytopenias. She was treated with cyclophosphamide, vincristine, and valganciclovir during that admission and was continued on this regimen along with rituximab as an outpatient.

Over the next several months, she developed worsening left upper quadrant abdominal pain, anemia, and thrombocytopenia, which were attributed to progressive splenomegaly. She had hepatosplenomegaly seen on imaging since at least 2010, but she was asymptomatic from this until 2014. She underwent a laparoscopic splenectomy in May 2014, with subsequent improvement in her pain and cytopenias. The histology of the spleen showed extended red pulp due to congestion, fibrosis, and focal extramedullary hematopoiesis. There was no evidence of MCD, KS, or infectious mononucleosis (i.e., EBV) noted in the specimen.

**Figure 2:**
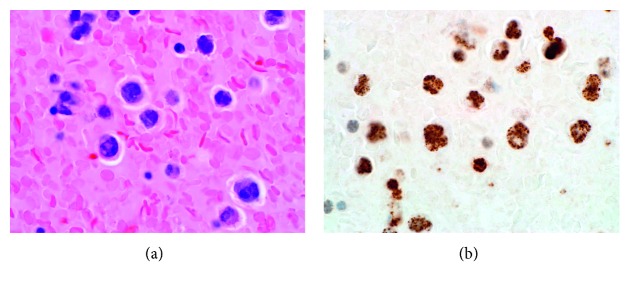
Primary effusion lymphoma, cell block preparation. (a) Large atypical cells with irregular nuclei and visible nucleoli (H&E, 1000x). (b) The large cells in the pleural effusion are positive for HHV-8 (immunostaining, anti-HHV-8 (13B10) mouse monoclonal antibody by Cell Marque, 1000x).

In June 2014, she was readmitted to our hospital, this time for progressive dyspnea. She was diagnosed with nonischemic heart failure with reduced ejection fraction. Her CD4 count was 746 cells/*μ*L, and her HIV viral load was undetectable (i.e., <20 copies/mL). She had a large right pleural effusion, which was drained via thoracentesis. Analysis showed this to be a malignant pleural effusion, which contained abundant large plasmablasts, positive with CD138, MUM1, CD30, HHV-8 LANA immunostaining, and lambda-light chain restricted by in situ hybridization, but negative for CD45, CD20, PAX5, CD79a, and CD3, consistent with PEL (see Figure 2). She was treated with a dose-adjusted regimen of etoposide, prednisolone, vincristine, and cyclophosphamide (EPOCH minus doxorubicin due to history of heart failure) for eight cycles, leading to a complete response.

As of most recent follow-up in March 2019, the patient is alive and remains in complete remission based on positive emission tomography (PET) results, resolution of her symptoms, and normalization of her laboratory testing. To the best of our knowledge, the patient has been maintained on ART since her HIV diagnosis without significant interruptions in treatment or the development of opportunistic infections. There was no apparent testing performed to assess for HHV-8 viremia, although there was a positive serum HHV-8 antibody (IgG) result in April 2014.

## 3. Discussion

Along with EBV, HHV-8 is classified into the gamma subfamily of Herpesviridae. Both DNA human oncoviruses utilize gene products which promote unregulated cell proliferation, particularly in immunosuppressed hosts. One of the key factors in the development of HHV-8-associated LPDs is the activation of the interleukin-6 receptor (IL-6R) signaling pathway, which, among other functions, promotes lymphoproliferation and angiogenesis [8, 9]. HHV-8-infected plasmablasts can be identified by their expression of the HHV-8 gene product known as LANA, a protein which may also function to increase the expression of human IL-6 (hIL-6) [10]. HHV-8 also encodes a functional homolog of hIL-6 called viral interleukin-6 (vIL-6), which can also activate the IL-6R but without the need for its coreceptor, gp80 [11].

Like other virally mediated lymphomas, HHV-8 potentiates an antiapoptotic effect via its constitutive activation of the family of transcription factors known as nuclear factor-kappa B (NF-*κ*B) [12]. In latently infected tumour cells, HHV-8 activates NF-*κ*B through its gene product known as viral FLICE inhibitory protein (vFLIP) [13]. Inhibition of NF-*κ*B has been shown to induce apoptosis in PEL [14]. A full review of the known or proposed mechanisms through which HHV-8 exerts its oncogenic effects is beyond the scope of this article and is covered in detail elsewhere [15].

HHV-8-associated LPDs show key features that distinguish one from another in most cases (see Table 1). Our patient met the typical features seen with HHV-8-positive MCD, PEL, and GLPD, with the exception that GLPD is usually seen in HIV-negative patients. However, cases of HHV-8-positive GLPD have previously been described in patients with HIV [6].

The identification of HHV-8 via staining for the nuclear antigen LANA is crucial for making the correct diagnosis. It helps to distinguish the HHV-8-associated LPDs from similar-appearing HHV-8-negative malignancies such as plasmablastic lymphoma and HHV-8-negative MCD. Assessing for the presence of EBER can also be helpful; it tends to be positive in GLPD and PEL and negative in MCD and DLBCL. It is uncertain what role, if any, that EBV has in the pathogenesis of GLPD and PEL, as cases of each condition have been described in EBV-negative patients [6, 16].

Diagnostic challenges can arise when observed cases do not neatly fit into one of the WHO-defined conditions on the HHV-8-associated LPD spectrum. Examples include a case of HHV-8^+^/EBV^+^ DLBCL, NOS, and another of HHV-8^+^/EBV^+^ MCD [17, 18]. The rarity of the HHV-8-associated LPDs likely contributes to the fact that the currently recognized entities fail to capture the full spectrum of reported cases. This leaves room for more detailed characterization of these disorders in future classification systems.

Many unanswered questions remain regarding our understanding of the spectrum of HHV-8-associated LPDs. For example, what causes an HHV-8-infected patient to develop one disease entity on this spectrum and not another? Why do some patients, such as the one we report here, develop multiple HHV-8-associated LPDs? The sequential development in our patient of a lambda-restricted MCD followed by a lambda-restricted PEL leads one to question whether there is a clonal relationship between these two entities or if they developed independently under the influence of HHV-8. Given their rarity, the creation of a biobank for HHV-8-associated LPDs would be a reasonable step to facilitate research in this area and to help answer these lingering questions.

Our patient has maintained a complete response from her three HHV-8-associated LPDs for five years now, since she was diagnosed with PEL. Her treatment regimen for PEL followed current guidelines of the National Comprehensive Cancer Network, which includes treatment recommendations for HHV-8-positive MCD, PEL, and DLBCL, NOS [19]. However, in its current iteration, it does not cover treatment for HHV-8-positive GLPD, which generally has a favorable prognosis when treated with chemotherapy or surgical resection with or without radiotherapy [20]. Clinical experience with GLPD is extremely limited as only about 20 cases have been published in the literature to date.

As previously noted, immunocompromise (often but not exclusively due to HIV infection) is a significant risk factor that enhances the virulence of HHV-8 in susceptible hosts. Antiretroviral therapy is a critical component of treatment for HIV-positive patients diagnosed with HHV-8-associated LPDs, as it has been shown to improve survival in this cohort [21]. ART is generally not given as sole treatment of HHV-8-associated LPDs, although this may occur when patients are otherwise not good candidates for chemotherapy. Rituximab therapy should be considered in any HHV-8-associated LPD with CD20 positivity.

## 4. Conclusion

The HHV-8-associated LPDs represent a spectrum of disorders which can range from reactive lymphoid hyperplasia to aggressive lymphomas. Except for GLPD, they are usually seen in immunosuppressed patients, who appear to be more susceptible to the host of oncogenic factors employed by HHV-8. Identification of HHV-8 and other key features are critical to establishing the correct diagnosis, with the understanding that a minority of cases may not be easily characterized due to our evolving understanding of these disorders.

## Figures and Tables

**Table 1:** Typical features of the HHV-8-associated lymphoproliferative diseases.

Feature	DLBCL, NOS	GLPD	MCD	PEL
Clinical presentation	Diffuse LAN, constitutional symptoms, splenomegaly HHV-8 MCD usually present	Localized LAN	Diffuse LAN, constitutional symptoms, splenomegaly	Serous effusions, no LAN
Immunosuppression	+/–	–	+/–	+/–
Microscopy	Sheets of PB/IB, effacement of nodal architecture	PB/IB in germinal centers, nodal architecture intact	PB/IB in mantle zones Interfollicular plasmacytosis	PB/IB in serous effusions (esp. pleural, pericardial, peritoneal)
Phenotype	CD20 +/–, CD138 –	CD20 –, CD138 –	CD20 +/–, CD138 –	CD20 –, CD138 +/–
Clonality	Monoclonal	Poly- or oligoclonal	Polyclonal	Monoclonal
Ig	IgM *ƛ* +	Monotypic *κ* or *ƛ*, any heavy chain	IgM *ƛ* +	–
HHV-8 (LANA)	+	+	+	+
EBV (EBER)	–	+	–	+
Prognosis	Poor	Good	Poor, but improving	Poor
Treatment	EPOCH or CHOP plus rituximab if CD20+, ART if HIV+	Chemotherapy (e.g., CHOP) or surgical resection +/–RT, ART if HIV+	No organ failure: rituximab Organ failure: chemotherapy and/or rituximab ART if HIV+	EPOCH or CHOP plus rituximab if CD20+, ART if HIV+

CD: cluster of differentiation; CHOP: cyclophosphamide, doxorubicin, vincristine, prednisone; EPOCH: etoposide, prednisolone, vincristine, cyclophosphamide, doxorubicin; IB: immunoblast; Ig: immunoglobulin; LAN: lymphadenopathy; PB: plasmablast; RT: radiation therapy.

## Abbreviations

AIDS: Acquired immunodeficiency syndrome
ART: Antiretroviral therapy
CHOP: Cyclophosphamide, doxorubicin, vincristine, prednisone
DLBCL, NOS: Diffuse large B-cell lymphoma, not otherwise specified
EBV: Epstein–Barr virus
EPOCH: Etoposide, prednisolone, vincristine, cyclophosphamide, doxorubicin
GLPD: Germinotropic lymphoproliferative disorder
HHV-8: Human herpesvirus 8
hIL-6: Human interleukin-6
HIV: Human immunodeficiency virus
KS: Kaposi's sarcoma
LANA: Latency-associated nuclear antigen
LPD: Lymphoproliferative disorder
MCD: Multicentric Castleman's disease
PEL: Primary effusion lymphoma
WHO: World Health Organization.
